# Polyphenolic Antioxidants in Lipid Emulsions: Partitioning Effects and Interfacial Phenomena

**DOI:** 10.3390/foods10030539

**Published:** 2021-03-05

**Authors:** Marlene Costa, Sonia Losada-Barreiro, Fátima Paiva-Martins, Carlos Bravo-Díaz

**Affiliations:** 1 REQUIMTE-LAQV, Departamento de Química e Bioquímica, Faculdade de Ciências, Universidade do Porto, 4169-007 Porto, Portugal; marlene.andreia.costa@gmail.com (M.C.); sonia@uvigo.es (S.L.-B.); mpmartin@fc.up.pt (F.P.-M.); 2 Department of Physical Chemistry, Faculty of Chemistry, Universidad de Vigo, 36200 Vigo, Spain

**Keywords:** antioxidants, emulsions, partitioning, lipid oxidation

## Abstract

The autoxidation of lipids in complex systems such as emulsions or biological membranes, although known to occur readily and to be associated with important pathological events, is lacking in quantitative data in spite of the huge efforts that have been made in attempting to unravel the complex mechanisms of lipid oxidation and its inhibition by antioxidants. Lipids are present as oil-in-water emulsions in many foods and pharmaceutical formulations, and the prevalent role of the interfacial region is critical to understand the antioxidant behavior and to correctly interpret antioxidant efficiencies. The aim of this review is to summarize the current knowledge on the chemical fate of antioxidants before they react with peroxyl radicals. Many researchers highlighted the predominant role of interfaces, and although some attempts have been made to understand their role, in most instances, they were essentially qualitative and based on putative hypotheses. It is only recently that quantitative reports have been published. Indeed, knowledge on the effects of relevant experimental variables on the effective concentrations of antioxidants is necessary for a successful design of alternate, effective antioxidative solutions.

## 1. Introduction

Fats are important components of cell membranes constituting a major source of energy for the human body [1,2]. They are carriers for nutrients such as fat-soluble vitamins, which are precursors of bioactive compounds that are involved in many vital processes [1,3,4].

**Scheme 1:**
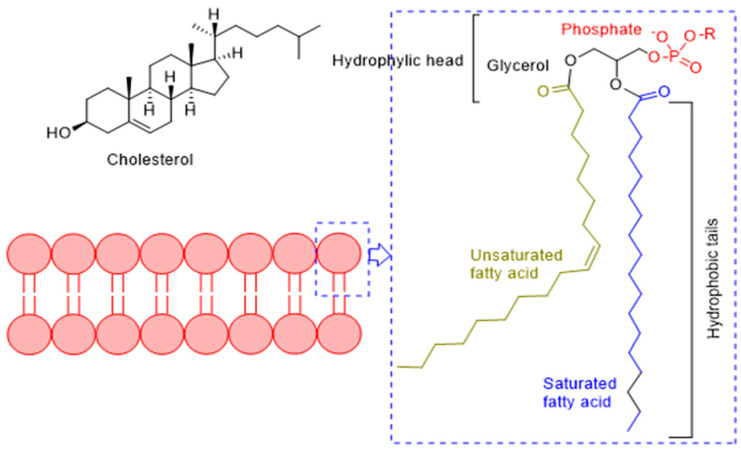
Chemical structures of cholesterol and a phospholipid and illustrative representation of a phospholipid bilayer.

More than 90% of our fat lipids are triacylglycerols (TAGs), which are commonly long-chain fatty acyl esters of glycerol (Table 1). In addition to being the major source of energy for the human body, dietary fat is the body’s only source of the essential fatty acids alpha-linolenic acid (an omega-3 fatty acid) and linoleic acid (an omega-6 fatty acid). These acids are precursors of several important molecules such as eicosanoids (affecting inflammation and many other cellular functions), the endocannabinoids (affecting mood, behavior, and inflammation), the lipoxins and resolvins, isofurans, neurofurans, isoprostanes, hepoxilins, epoxyeicosatrienoic acids (EETs), and neuroprotectin D. They also affect cellular signaling and control transcriptional factors. The typical Western diet also contains unesterified cholesterol, which is derived from cell membranes present in foods of animal origin. Usually, esterified cholesterol can only be found in liver or food manufactured with blood. While cholesterol, Scheme 1, does not provide energy, it is usually incorporated in the group of dietary fats because of its relevant role in various metabolic processes, including the synthesis of a number of hormones, bile acids, and Vitamin D. However, its intake needs a strict control because of health implications, and its presence in the food supplies is basically restricted to fats of animal origin [2].

Approximately 5% of dietary lipids are phospholipids from cell membranes. These molecules have a glycerol backbone moiety esterified at the first two positions to long-chain fatty acids and at the third position to a phosphate that is also esterified to a polar group, usually choline, serine, or ethanolamine. The shape and amphipathic nature of phospholipids makes them to form bilayers spontaneously in aqueous environments (Scheme 1).

## 2. Lipid Intake: Health and Nutritional Aspects

In view of the chemical structures of the fatty acids shown in Table 1, one can easily deduce that they do not dissolve easily in water, and nature provided imaginative solutions to overcome the problem of lipid transport in the blood to the target tissues [5]. Knowing these imaginative solutions is a way to understand many phenomena that happen in emulsified systems and how to improve these systems.

**Figure 1:**
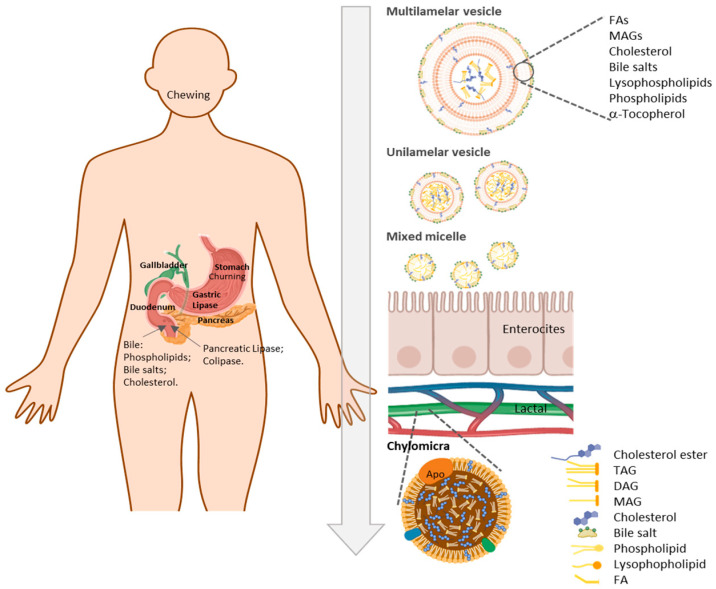
Illustrative steps of the digestion process of fats into the digestive track. Breakdown of multilamellar emulsion droplets to mixed micelles. The core of the emulsion vesicles contains triacylglycerols (TAGs), diacylglycerols (DAGs), and cholesteryl esters. On the surface are fatty acids, MAGs, phospholipids, lysolecithins, and cholesterol. Initially, gastric lipase is adsorbed to the surface, which is followed by pancreatic lipase and colipase. The addition of more bile salts to the multilamellar vesicle converts them into unilamellar vesicles. The further addition of bile salts leads to the formation of a mixed micelle. Mixed micelles carry lipids to the enterocyte surface and enter the enterocyte by nonionic diffusion, incorporation into the membrane (“collision”), or by carrier-mediated transport. The re-esterification of digested lipids by the enterocyte leads to the formation and secretion of chylomicrons to the lymphatic system.

The central event in the digestion of lipids is their hydrolysis catalyzed by lipases in the aqueous media present at the intestinal lumen [6,7]. Since dietary lipids are insoluble in water, lipases have to act at oil–water interfaces. Therefore, the first step in the lipid digestion is the transformation of ingested fats into a fine oil-in-water emulsion. This emulsification begins with food preparation, followed by chewing and gastric churning. The bile secreted into the duodenum plays a key role in stabilizing the fine emulsion produced by coating the emulsion droplets with phospholipids, bile salts, and unesterified cholesterol [6,7], as shown in Figure 1, and it prevents lipid vesicles from coalescing. Moreover, some digestion products, such as fatty acids (FAs) and monoacylglycerols (MAGs), and vitamin E (α-tocopherol) or food emulsifiers also take part of this coating. The droplet core is mainly composed of TAG, which also contains cholesteryl esters and other nonpolar or weakly polar compounds such as carotenoids and xanthophiles. Nevertheless, a small amount of TAGs is present at the surface of the amphiphilic coating [7].

Fat digestion takes place by action of gastric lipase and by the secretion of pancreatic enzymes, including colipase-dependent pancreatic lipase and esterases, with the release of two fatty acids and a single 2-monoacylglycerol (2-MAG) [8]. The pancreas also secretes carboxyl ester hydrolase, which is active on a wide range of esters, and phospholipase A_2_ (PLA_2_), which is active on glycerophospholipids, releasing usually an unsaturated fatty acid and a 1-lysophospholipid [8].

Then, the lipolytic products also act as emulsifiers, and as surface TAGs are hydrolyzed, they are replaced by TAGs from the core of emulsion droplets. As these droplets become progressively smaller and the concentration of surfactant digestion products increases, there is an increase in the surface area and, as a consequence, an increase in the rate of hydrolysis. During this process, bile-salt transform the initial multilamellar vesicles into unilamellar vesicles and then into mixed micelles composed of bile salts and mixed lipids (MAG, lysophospholipids, fatty acids, and cholesterol) [8], as shown in Figure 1.

When the mixed micelles reach the enterocyte apical surface, they find an acidic microclimate where the fatty acids are now protonated and can leave the micelle to enter the enterocyte. The uptake of fatty acids can now occur by nonionic diffusion or by incorporation into the enterocyte. However, it is clear that at least three integral membrane proteins promote the uptake of fatty acids. Unesterified cholesterol, isophospholipids, and 2-MAGs also leave the micelle carrier and enter the enterocyte as monomers by simple diffusion across the apical membrane or by a carrier-mediated transport. After absorbing FAs, lysophospholipids, MAGs, and cholesterol, the enterocyte re-esterifies them and assembles the esterified products with specific apoproteins into emulsion-like particles called chylomicrons (Figure 1). Chylomicrons are lipoprotein particles that consist of a core of triglycerides and cholesterol esters (−90%) coated by a monolayer of phospholipids (−10%) and cholesterol (1–3%) where proteins (1–2%) are embedded, and they transport dietary lipids from the intestine through the lymph for ultimate delivery to other organs via the bloodstream. This process enables the delivery of dietary lipids and liposoluble vitamins to their intended target tissues (muscle and adipose tissue) [8].

### 2.1. Lipids in Enteral and Parenteral Nutrition

Life requires a constant input and output of energy, allowing the body to perform daily functions and tasks such as breathing, walking up a flight of steps, and even reading this manuscript. Energy is usually obtained through the intake of various foods, and a large portion of a person’s energetic requirements are provided by the lipids contained in the food, which in most cases are present as oil-in-water emulsions [9,10]. Milk is representative of a naturally occurring food emulsion, but there are many other manufactured food emulsions such as mayonnaise, salad dressings, creams, etc.

Energetic requirements can be met by feeding people with carbohydrates and protein alone, but a lack of fat can result in essential fatty acid deficiency [10,11]. Therefore, in situations where energy supply via the enteral route cannot cover the necessary needs of the human body (for example, in intestinal failure), parenteral nutrition—referred to the delivery of nutrients into a vein—is a life-saving procedure used all over the world [12,13,14,15,16,17,18].

Lipids provided intravenously do not go through the intestinal lumen and are not hydrolyzed by pancreatic lipases, emulsified by bile salts, or packed into chylomicrons as dietary fats. Thus, these lipids must be provided in a similar fashion to chylomicrons to empower them to be transferred into the hydrophilic physiological environment, i.e., they are provided as small droplets dispersed in an aqueous carrier (oil-in-water emulsions) [11,15,18].

Parenteral preparations can be as simple as carbohydrate intravenous solution to the much more sophisticated parenteral emulsions employed to feed infants, children, and adults. The lipids in these parenteral products not only have a high energetic value and low osmolality, but they also prevent the complications of using glucose as the only non-protein energy source such as hyperglycemia, fatty acid deficiency, and hepatic steatosis [19]. Therefore, all these formulations need to include lipids among their components, as shown in Table 2, because if absent, there is a serious risk of growth impairment, dermatitis, and renal and pulmonary diseases, among others [10].

The first well-tolerated lipid emulsion (intralipid) used soybean oil (SO) rich in omega-6 polyunsaturated fatty acids (ω-6 PUFAs) [20]. However, high ω-6 PUFA administration has been associated with oxidative stress and inflammation [21]. Therefore, lipid emulsions containing pure fish oil (FO), pure olive oil (OO), or various blends of medium-chain triacylglycerols, SO, OO, and FO have been available. The introduction of FO into nutrition mixtures had several beneficial *in vivo* effects due to their immunomodulatory and anti-inflammatory activity [22,23,24] and also as hepatoprotective. However, omega-3 polyunsaturated fatty acids (ω-3 PUFAs) are susceptible to lipid peroxidation due to the high number of double bonds present in their fatty acids structure and may increase the risk of oxidative stress [23]. In fact, oxidative stress is proposed to be one of the processes that leads to several diseases of premature infants, as they have limited antioxidant system and are exposed to more oxygen, ventilation, and infections [25].

In addition to lipids and water, food emulsions are formulated with a variety of other ingredients including surfactants, proteins, carbohydrates, vitamins etc., to tailor the bulk physicochemical properties of the food emulsion (nutritional properties, appearance, texture, and both physical and chemical stability). α-Tocopherol is the main antioxidant in biomembranes and has the dual role of antioxidant, preventing the oxidation of parenteral emulsions, and vitamin. Lipid-based emulsions can be also employed to deliver lipophilic and amphiphilic bioactives and other antioxidants [11,12,26,27,28].

### 2.2. Nutritional Consequences of Lipid Peroxidation

Lipid peroxidation is a chemical reaction that basically involves the uptake of an H atom from a lipid molecule, generating a lipid radical [29,30]. Further reaction of the lipid radical with oxygen in the air generates peroxyl and hydroperoxyl lipid radicals, which can be quenched by reaction with other radicals or by reaction by radical scavengers or chain breakers (e.g., antioxidants) or can further react to produce harmful products and non-radical products [30,31,32]. The reaction occurs naturally in the human body as a part of several metabolic pathways or because the lipid-containing tissues are, unavoidably, in contact with molecular oxygen or with reactive oxygen and nitrogen species (ROS and RNS, respectively). Table 3 shows the main ROS species [33,34]. Since the radicals produced in the course of the reaction are extremely reactive, they react with any molecule in their vicinity including other fatty acids, amino acids and proteins, and endogenous antioxidants [35]. As a consequence, the structural integrity of cells may break down if the natural antioxidant defense is not working properly and a number of diseases may be boosted, including neurodegenerative diseases, cancer, cataract, diabetes, and atherosclerosis, among others [35,36].

In lipid-based food products, the reaction may be boosted in the presence of radical initiators such as light, ionizing radiation, or metals. No matter how lipid peroxidation is initiated, the immediate consequence of the reaction is the deterioration of foods, a significant decrease in the quality of the food product, and the production of bad odors and smells (rancidity) [37]. The development of off-flavors and aromas as well as deterioration in appearance takes place long before concentrations of lipid oxidation products have reached toxic levels but it largely affects the consumer´s confidence and trust in the product [29,32]. However, nutritionists have been concerned with the potential adverse effects of lipid oxidation in foods because of the reduction of the nutritive value of foods components and of the toxicity of oxidation products [37,38].

The reaction is also of a great concern in medicine, because significant adverse effects may also occur through the interaction of lipid radicals or lipid oxidation products with proteins and vitamins, as they can react with amino acids in proteins to form carbon-centered protein radicals and cause protein denaturation and protein scission, so that these oxidative processes produce subsequent damage to surrounding tissues in the human body and protein malfunction that is believed to be closely associated to aging and to various neurodegenerative diseases [35,36,39].

### 2.3. Kinetics and Mechanisms of Lipid Peroxidation in Brief

The autoxidation of polyunsaturated fatty acids has received much attention due to its involvement in food spoilage and because of its relevance *in vivo* to membrane damage and in various diseases. The kinetics and basic mechanism of the reaction in bulk solution is fairly well known but, in spite of the thousands of publications on the topic, some aspects are still a matter of debate [40,41,42]. Here, we will just provide a brief background and will show the most important kinetic equations.

**Scheme 2:**
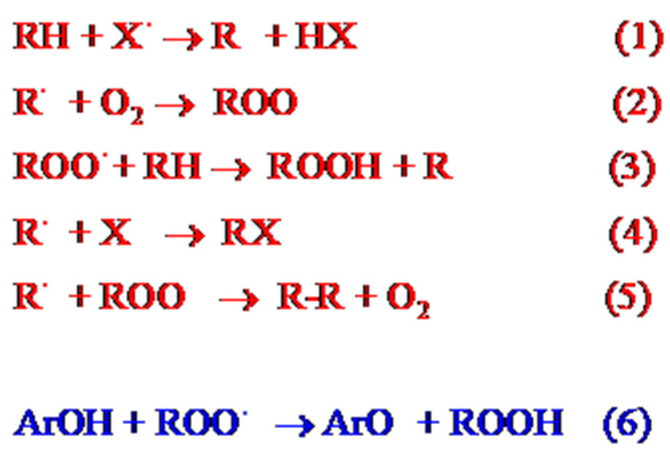
Main chemical reactions involved in the peroxidation of lipids (RH = lipid, X^•^= external free radical, R^•^ = lipid radical, ROO^•^ = peroxyl radical, ROOH = lipid hydroperoxide, ArOH = antioxidant).

Kinetic modeling of a reaction is useful for elucidating aspects of complex chemistry, especially in biological systems, and instructive models of liposomal, mitochondrial, and cellular lipid oxidation have highlighted particular characteristics of these multiphase systems, in particular the compartmentalization of these structures with different solvation properties. A brief summary of the kinetic model is provided, and the interested reader is referred to specialized publications [31,41,42,43,44,45,46,47]. Relevant reactions involved during oxidation are outlined in Scheme 2.

The reaction starts with the formation of a lipid radical R^•^ from an external free radical X^•^ that may be formed spontaneously (e.g., with the aid of thermal energy) or induced by radiation, metals or—as in most kinetic studies—through the addition of a suitable azo dye radical initiator (reaction 1, Scheme 2) [29,30]. The initial lipid radical reacts rapidly with molecular oxygen to give a peroxyl radical ROO^•^ (reaction 2, Scheme 2) that, during the propagation step, reacts with another lipid molecule (reaction 3, Scheme 2) to yield a lipid hydroperoxide and a new lipid radical, which is introduced in a chain reaction, leading to the formation of a variety of products. The reaction ends when non-radical products are formed, which is usually after all the lipid substrate has been consumed and the radicals quench themselves (reactions 4 and 5 in Scheme 2) [48,49,50].

The lipid oxidation reaction can be minimized when antioxidants (ArOH) are present, as shown in reaction 6 in Scheme 2. Antioxidants are molecules that, basically, are capable of donating an H-atom to a lipid radical to regenerate the parent lipid molecule, yielding a much less reactive radical than can be quenched by reactions with other lipid or antioxidant radicals [30,43,50].

The reaction 2 (Scheme 2) between the formed carbon-centered radicals with molecular oxygen is fast and essentially diffusion controlled, and the rate of oxygen uptake is given by Equation (1), where *k*_p_ is the propagation rate constant [49,51].

(1) $$-\frac{d[O_{2}]}{dt}=k_{p}[ROO^{•}][R-H]$$

The rate of chain initiation is usually controlled and known because a suitable initiator (e.g., R-N=N-R) with a known rate of decomposition, *k*_i,_ is used. Since radicals may not “escape” from the solvent cage in which they are formed to react with oxygen, an efficiency parameter *n* needs to be taken into consideration. Assuming the steady-state condition, the rate of chain initiation equals to that of termination, Equation (2), where *k*_t_ is the termination rate constant [41,49,50].

(2) $$r_{i}=2k_{i}n[R-N=N-R]=2k_{t}{{[\mathrm{ROO}}^{•}]}^{2}$$

Substituting for the reactive intermediate [ROO^•^] into Equation (1) gives Equation (3), which is the general mathematical expression for the uninhibited oxygen consumption.

(3) $$-\frac{d[O_{2}]}{dt}=\frac{k_{p}}{2k_{t}^{1/2}}[R-H]r_{i}^{1/2}$$

**Figure 2:**
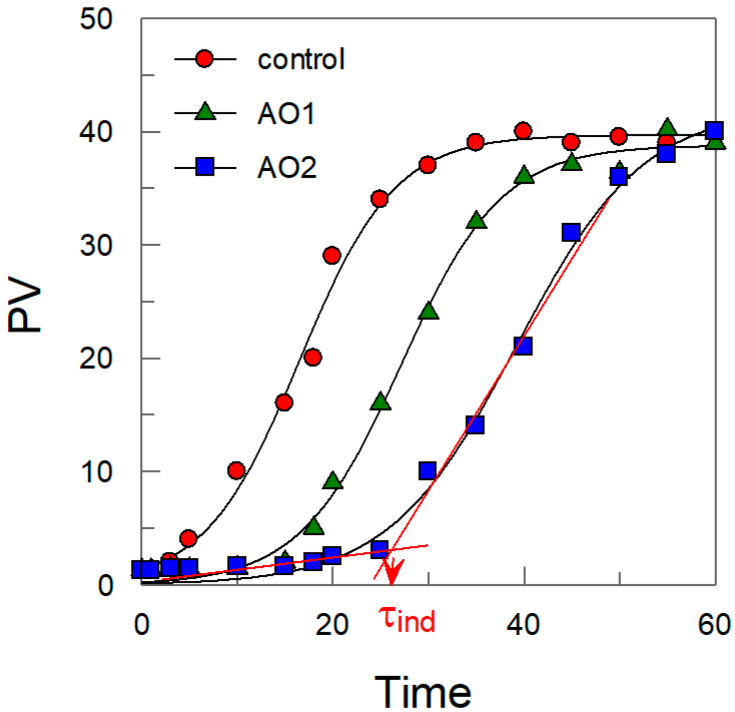
Illustrative plot of the kinetics of production of peroxidation products (PV) in the absence (control) and in the presence of two hypothetical antioxidants AO1 and AO2 under the same experimental conditions and determination of the induction time τ_IND_. Both PV values and time are given in arbitrary units. Solid lines drawn to aid the eye.

The peroxidation reaction is usually inhibited when antioxidants (ArOH) are present, as shown in reaction 6 in Scheme 2. For quantitative kinetic determinations, the rate of chain initiation *r*_i_ should be determined, usually with the aid of an antioxidant ArOH, as shown in Equation (4):

(4) $$r_{i}=\frac{n[ArOH]}{\tau_{ind}}$$

where *τ_ind_* is determined graphically from the intercept of the slopes of the inhibited and uninhibited reactions, as illustrated in Figure 2 [50].

## 3. Antioxidant Defense against Lipid Peroxidation

Recent cohort human epidemiological studies support the reported inverse relationships between antioxidants (AOs) intake and the development of neurodegenerative diseases [52,53]. In addition, polyphenol-rich herbs and spices have been used for years in natural and preventive medicine.

Polyphenolic antioxidants are mostly obtained from natural resources (fruits, vegetables, etc.), and they are widely employed to delay or prevent the oxidation of biomolecules and in the attempt to protect cellular components from the oxidative damage caused by ROS and transition metals [54,55,56,57,58]. However, within certain conditions, they can also exert a dangerous pro-oxidant action, with the production of both pro-oxidant catalysts and free radicals. However, this pro-oxidant activity may also be essential for the polyphenols’ capacity to work as antimicrobial and antipathogenic agents in immunological defense mechanisms and aging processes. Such activity depends on the physiological conditions, polyphenol chemical properties, and on their distribution within the biological system.

**Scheme 3:**
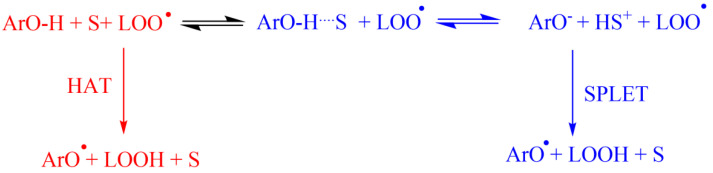
Hydrogen atom transfer (HAT) and sequential proton-loss electron transfer (SPLET) mechanisms. Both mechanisms are competitive with each other, and although the rate constant for the electron transfer in SPLET is much higher than the rate constant for hydrogen transfer in HAT, even small amounts of phenoxide anions ArO^−^ can make the SPLET to dominate over HAT [41]. ArOH: antioxidant, LOO^•^: peroxyl radical, S: solvent.

Antioxidants are usually classified as primary or “chain-breaking” antioxidants and are free radical acceptor molecules that delay, inhibit, or interrupt the propagation step by trapping lipid radicals once the peroxidation chain has started. Primary antioxidants quench free radicals through four main mechanisms: (1) transferring H-atoms to peroxyl radicals (hydrogen atom transfer, HAT, mechanism); (2) single electron transfer-proton transfer (SETPT mechanism); (3) sequential proton loss-electron transfer (SPLET) mechanism and (4) electron transfer–proton transfer (ETPT) [41,59]. Scheme 3 illustrate the most common HAT and SPLET mechanisms.

**Scheme 4:**
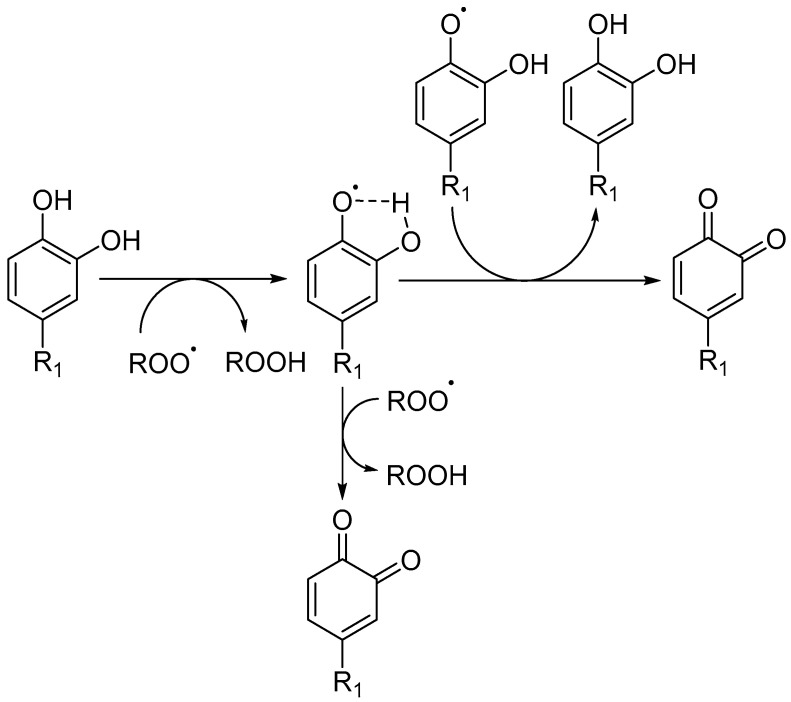
Radical scavenging activity of *o*-chatechols: initial semiquinoid radical produced can be further oxidized to a quinone.

Primary antioxidants include mainly mono- and polyhydroxy phenols with various ring substituents in different positions. Phenol itself is inactive, but the substitution of alkyl groups into the 2, 4, or 6 positions increases the electron density on the hydroxyl group by an inductive effect and, thus, increasing their reactivity with lipid radicals. On the other hand, when these alkyl groups are bulky, the reactivity of the radical formed decreases by steric hindrance. The antioxidant effectiveness is directly related to the resonance stabilization of the phenoxyl radicals, and therefore, the introduction of a second hydroxyl group into the 2 or 4 position of a phenol (catechols) increases the antioxidant activity because the initial semiquinoid radical produced can be further oxidized to a quinone by the reaction with another lipid radical, as shown in Scheme 4.

On the one hand, this increase in the antioxidant activity is due to the possibility of the radical formed being stabilized by a hydrogen bridge with the second hydroxyl group and, on the other hand, to the ability of compounds to supply one more hydrogen that can neutralize another radical, with the production of a non-radical quinone, and therefore make it non-reactive [50].

Examples of primary antioxidants include several natural molecules such as tocopherols (vitamin E) and numerous flavonoids and phenolic acids as well as synthetic antioxidants such as butylated hydroxyanisole, propyl gallate, and *terc*-butylhydroquinone.

Secondary (or preventive) antioxidants are molecules that are capable of preventing radicals formation or inactivating radicals before they enter into the radical propagation step. Preventive antioxidants usually include complex enzymatic molecules such as catalase, superoxide dismutase, and glutathione peroxidase and metal chelators, as shown in Table 4. These antioxidants are usually endogenous in cellular systems and contribute to minimizing the effects of ROS. Some primary and preventive antioxidants are synergists because they are able to regenerate primary antioxidants consumed during the inhibition reaction. Citric acid, ascorbic acid, ascorbyl palmitate, and lecithin are good examples of synergistic antioxidants.

Sometimes, a third group of antioxidants is considered. These tertiary antioxidants are usually co-factors of enzymes involved in repairing processes of the damage caused by the lipid radicals to proteins and DNA, and they include zinc, folates, and cobalamin (vitamin B12).

### Antioxidants in Delivery Systems

The human body produces endogenous antioxidants as a defense to ROS, but sometimes, the concentration of antioxidants in the plasma is below the minimum effective concentration and, thus, it is necessary to have supplementation from external sources, for instance from foods or antioxidant supplements.

**Scheme 5:**
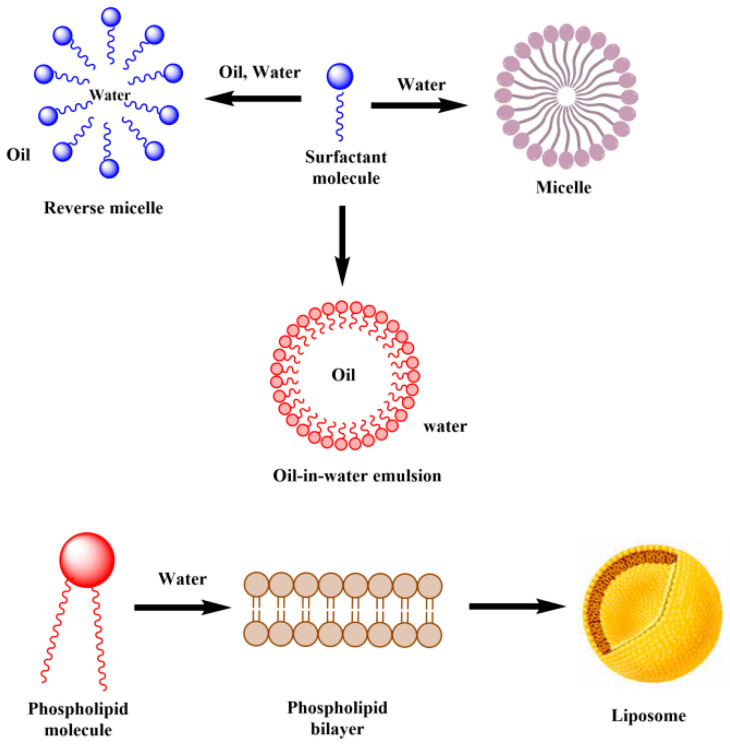
Some examples of surfactant-based encapsulation systems.

There are several ways of encapsulating antioxidants, as shown in Scheme 5, and the choice of the delivery system is crucial for the practical applications of both the delivery system and the encapsulated material. Among them, the lipid or surfactant-based systems are mostly employed because they are biocompatible, have low preparation and modification costs, and are easy to scale up [61,62].

Among all antioxidant delivery systems, lipid emulsions are commonly used in a variety of food and pharmaceutical preparations because of their biocompatibility, biodegradability, relative stability, very convenient handling, and cost-effective manufacturing processes [9,10]. They are modern colloidal drug carriers, currently used as parenteral formulations, to improve analgesic therapy and to deliver water-insoluble drugs, and for dermal and transdermal applications.

## 4. Lipid-Based Emulsions: Preparation, Physical Stability, and Domains

Since the free energy needed to increase the surface area between the oil and water phases is positive, a direct contact between two immiscible liquids (e.g., water and oil) is energetically highly unfavorable. As a consequence, emulsions are inherently thermodynamically unstable systems (as opposed to microemulsions). When mixed, both liquids have the tendency to minimize their common area, eventually resulting in phase separation with the oil (lower density) on top of the aqueous phase. In fact, the physical stability of an oil–water mixture is not very high, and a variety of phenomena including flocculation, creaming, coalescence, and Ostwald ripening may occur [63]. To form kinetically stable emulsions for longer periods of time (hours to years), surfactants are added before homogenization. These molecules adsorb at the oil–water interface, creating a protective coating that prevents droplets from aggregating. Typical emulsifiers include small molecule surfactants, proteins, and phospholipids.

**Scheme 6:**
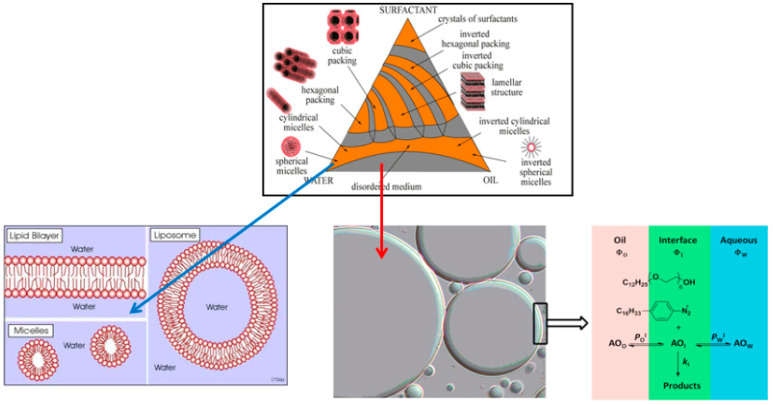
Hypothetical pseudoternary phase diagram for a three-component system of water (i.e., aqueous solutions that may contain buffers or salts), oil and surfactant, and images (not drawn to scale) of various physical structures present. The mesostructures are composed by a large number of molecules, so that that at the molecular level, in general, the surface curvatures are quite small, and in emulsions, they are essentially flat compared to the size of the surfactant molecules. Adapted from ref. Romsted, L. S. Introduction to Surfactant Self-Assembly; Wiley and Sons: New York, 2012; Vol. 1.

Scheme 6 is a pseudo-ternary phase diagram for a putative system composed of oil, water, and surfactant, and where the open areas represent homogeneous mesophases. Various structures can be formed depending on the amounts of surfactant, oil, and water employed. These structures have different aggregate shapes because of changes in the delicate balance of forces controlling their stabilities. No matter their shape, all of them share the same basic structural commonality: an interfacial region that separates oil and water regions. The interfacial region is highly anisotropic, showing a gradient of polarity that falls sharply from that of water to that of the oil.

Emulsions are usually classified according to the relative organization of the oil and aqueous phases as oil-in-water (o/w) emulsions (oil droplets dispersed in an aqueous continuous phase) and water-in-oil (w/o) emulsions (water droplets dispersed in the oil continuous phase). Various emulsion systems can currently be prepared, including micro-, nano-, self-emulsifying, dry, pickering, and multiple emulsions (e.g., oil-in-water-in-oil (o/w/o) or water-in-oil-in-water (w/o/w) type).

The relative physical stability of emulsified systems is commonly based on the analysis of different emulsion properties such as droplet size distribution, droplet concentration, droplet charge, and the nature and concentration of the emulsifier. Regardless of their nature and particle size, successful emulsions are usually evaluated in terms of their physicochemical properties and physical and chemical stability, but depending on their particular use, other parameters may also need to be taken into consideration. For example, formulations of emulsions designed for cosmetics require preventive microbial growth.

### 4.1. Distribution of Antioxidants in Emulsions

Antioxidants (AOs) are mainly added to food emulsions to minimize the oxidation of lipids, but they may also be added because of their healthy bioactivity. In this section, we will focus on the fate of antioxidants once they are added to emulsions to describe their location in the system. Their reactivity with lipid radicals and the kinetic consequences of their distribution have been analyzed in Section 2.3.

**Scheme 7:**
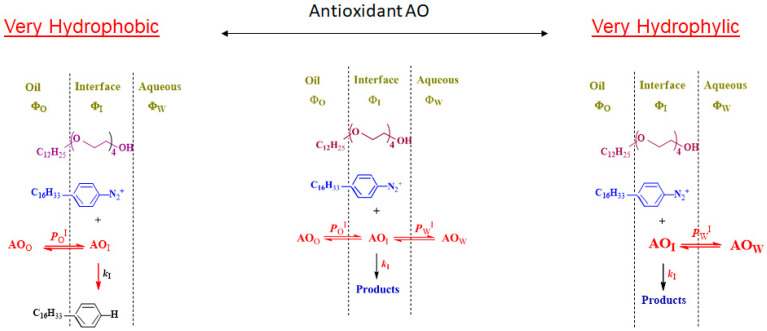
Conceptual division of the domains with different solvent properties and distribution of an antioxidant (AO) according to their polarity. Reproduced from S. Losada-Barreiro, PhD Thesis, Universidad de Vigo, 2014, with permission.

Conceptually, emulsions can be divided into three regions with distinct solvent properties: the core of droplets, the continuous phase, and the interfacial region (Scheme 7). The interfacial region can be described as a thin region surrounding each oil droplet, which is mainly composed of surface-active molecules but that also contains some oil, water, and other components that may be adsorbed in it (e.g., antioxidants, buffers). In fluid emulsions, all components are assumed to be transferred between the various regions at diffusion controlled—unless there are some physical restrictions. For instance, when antioxidants are removed in a given region (for example, after reacting with a peroxyl radical), they are replenished immediately by antioxidant molecules from other regions, so that diffusion is not limiting.

These operative assumptions permit defining antioxidant distributions in terms of equilibrium processes, so that when incorporated into the emulsions, they immediately distribute thermodynamically between the oil, interfacial, and aqueous regions. At equilibrium, the chemical potentials of the antioxidants in the distinct regions of the emulsion are equal to each other, and their distribution can be described by two partition constants, that between the oil and interfacial, *P*_O_^I^, and that between the water and interfacial, *P*_W_^I^, regions, as shown in Equations (5) and (6). Moreover, because the chemical potentials of the antioxidants only depend on the activity of the component in each region, the distribution of antioxidants does not depend on the size or shape of the droplets in the emulsion, as we have recently demonstrated [64,65].

(5) $$P_{W}^{I}=\frac{{(\mathrm{AO}}_{I})}{{(\mathrm{AO}}_{W})}$$

(6) $$P_{O}^{I}=\frac{{(\mathrm{AO}}_{I})}{{(\mathrm{AO}}_{O})}$$

In Equations (5) and (6), the concentrations given in parentheses are expressed in moles per liter of the interfacial (I), oil (O), and aqueous (W) regions. It is worth noting that the ratio *P*_W_^I^/*P*_O_^I^ is, numerically, equal to the partition constant in a binary oil–water system, as shown in Equation (7). Thus, binary oil–water mixtures can be considered as limit cases of oil-in-water systems where the concentration of surfactant is low enough to allow complete phase separation, i.e., when no surfactant is added.

(7) $$P_{W}^{O}=\frac{{(\mathrm{AO}}_{O})}{{(\mathrm{AO}}_{W})}$$

Two limiting situations can be envisaged, as shown in Scheme 7. When the antioxidant is very hydrophilic (e.g., phenolic acids, for example), antioxidants are essentially oil-insoluble so that their concentration in the oil (O) region is negligible, and only *P*_W_^I^ (Equation (5)) is needed to describe their distribution. Conversely, very hydrophobic AOs are water insoluble and only a partition between the interfacial (I) and oil (O) regions, and their distributions are described by the partition constant *P*_O_^I^, as shown in Equation (6) [44,45,66,67,68].

**Scheme 8:**
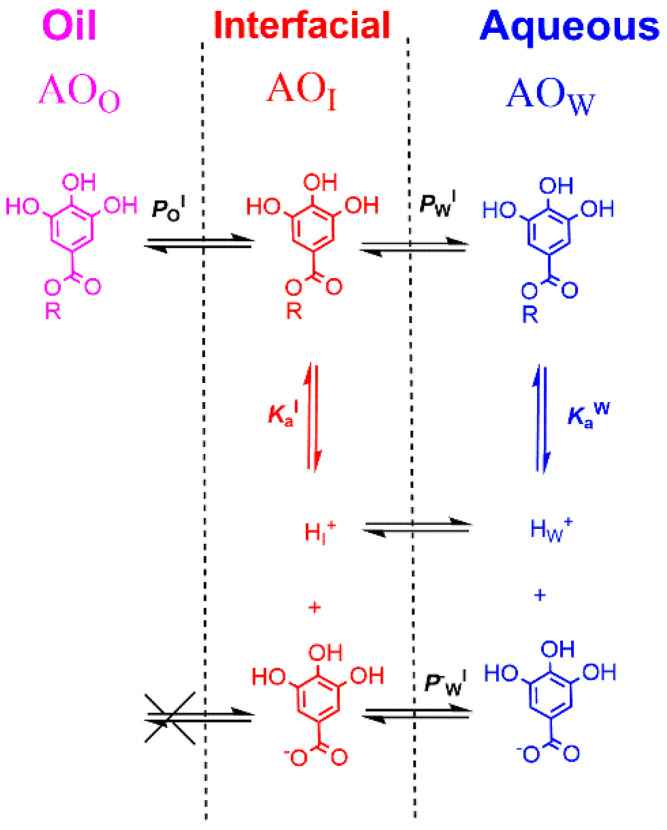
Equilibria for the distribution of gallic acid (GA) and its derivatives, in emulsions. Only GA (R = H) ionizes significantly at the typical acidities (pH = 2–6) of the emulsified foods. The alkyl derivatives (R = −C_n_H_(2n+1)_, n = 1, 2, …, 12) distribute in different extents between the three regions depending on their hydrophilic lipophilic balance.Adapted from ref. 102 with permission. Copyright Royal Society of Chemistry.

The distribution of AOs may also depend on the pH of the aqueous phase. If the AO ionizes (as may be the case, for example, of phenolic acids), its ionization equilibrium needs to be considered, as shown in Scheme 8. The concentration of ionized species in the oil region should be negligible, but both neutral and ionized species may coexist in the interfacial and aqueous regions. In this case, the partition constant value depends on the pH, and the apparent partition constants *P*_O_^I^ (app) and *P*_W_^I^(app) are given by Equations (8) and (9), respectively.

(8) $$P_{O}^{I}(\mathrm{app})=\frac{{(\mathrm{AO}}_{I}{)+(\mathrm{AO}}_{I}^{-})}{{(\mathrm{AO}}_{O})}$$

(9) $$P_{W}^{I}(\mathrm{app})=\frac{{(\mathrm{AO}}_{I}{)+(\mathrm{AO}}_{I}^{-})}{{(\mathrm{AO}}_{W}{)+(\mathrm{AO}}_{W}^{-})}$$

### 4.2. Importance of Determining Partition Constants in Binary Oil–Water Systems: Can They Be Envisaged from Octanol–Water Values?

The distribution of antioxidants in binary oil–water systems, and hence their partition constants values, as defined by Equation (7), depend on the balance of all intermolecular forces—electrostatic, hydrogen bonding, and dispersion interactions—between the solute and the phases in which they are dissolved [44,69,70]. Hence, one should expect that their values will depend on the solvent properties of the oil employed.

Knowledge of partition constant values in binary oil–water mixtures is of great interest for the following reasons:

(1) They can be used as a measure of the relative tendency of the antioxidant to be located in a specific region [71] because the Gibbs free energy, ΔG^O→W^, for the transfer of the antioxidant from the oil to the aqueous region depends on the magnitude of the *P*_w_^O^ values, as shown in Equation (10). Equations (7) and (10) are valid under the assumption that the solute concentration is low to consider activity coefficients of the solute close to the unit, so that it is safe to use concentrations instead of activities. Further details on these and other assumptions and involved equations can be found elsewhere [69,71,72].

(10) $$\Delta G^{W\to O}=-\mathrm{RT}\ln P_{W}^{O}$$

(2) The partition coefficient also involves the thermodynamics of the relative solvation of the solute between aqueous and the nonpolar phase in terms of binding affinities or hydration/solvation free energies.

(3) Knowledge of *P*_w_^O^ values describing the partitioning of a single solute in a given ionization state between two essentially immiscible liquids is of general interest because they are very useful to determine the partition constants in emulsified systems and, as indicated before, its value is numerically equal to the ratio of the partition constants in emulsified systems (Equations (5) and (6)).

**Scheme 9:**
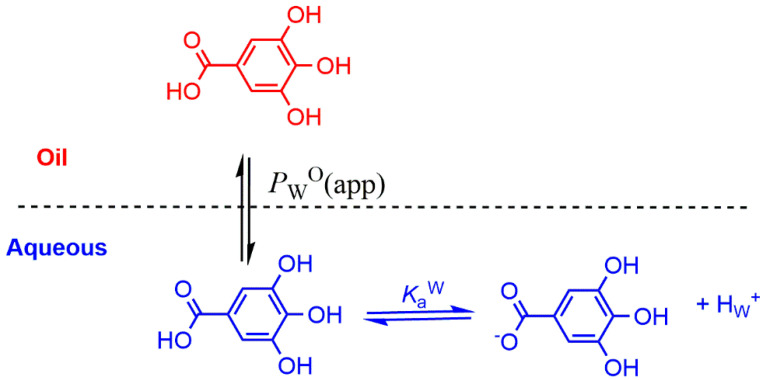
Partitioning of a representative phenolic acid antioxidant (gallic acid, GA) between oil and water phases. Appreciable ionization only takes place in the water phase. When GA is completely protonated, *P*_W_^O^(app) = *P*_W_^O^. Adapted from ref [74].

If the AO is partially ionized under the experimental conditions as illustrated in Scheme 9 for gallic acid (GA), then partition constants depend on pH, and their distribution is better described by an apparent partition constant, *P*_W_^O^(app), as shown in Equation (11), which is defined taking into consideration all ionized and neutral forms present at a given pH. The ionization constants of weak acids in oils are typically 5–6 orders of magnitude smaller than those in the aqueous phase [73], (i.e., p*K*_a_(O) >> p*K*_a_(W)), and thus, the antioxidant is completely neutral at the low pH limit, and *P*_W_^O^(app) becomes equal to *P*_W_^O^, as can be predicted from Equation (11).

(11) $$P_{W}^{O}(\mathrm{app})=\frac{{(\mathrm{AO}}_{O})}{{(\mathrm{AO}}_{W}{)+(\mathrm{AO}}_{W}^{-})}$$

In Equation (11), the activity coefficients of AO forms in each phase are assumed to be close to the unity, and this approximation usually holds because the concentrations of the AOs employed in experimental studies are generally small [69,70,75].

Table 5 lists the experimentally determined *P*_W_^O^ values for the C1–C12 gallate esters in olive oil–water mixtures. As expected, *P*_W_^O^ values increase upon increasing the alkyl chain length of the antioxidant. To a first approximation, GA and the C1–C12 gallates have two distinct regions within their molecules that influence their partitioning: a phenolic residue, a carboxylic acid group, or an alkyl ester tail containing a variable number of carbons. The phenolic moiety, which is common to all of them, makes a constant contribution to solubility in water and oil. However, increasing the number of C atoms in the tail increases the hydrophobicity of the AO and its solubility in oil. Thus, the higher the number of C atoms, the greater the fraction of AO in the oil region and the greater the *P*_W_^O^ values.

Partition constants for antioxidants in oil–water mixtures are scarce in the literature, and its determination, although experimentally simple, may be tedious. For this reason, in many reports, partition constant values are extrapolated from those obtained in octanol–water mixtures, *P*_W_^OCT^, which are frequently used as a measure of lipohilicity in drug discovery. For the sake of comparisons, the *P*_W_^OCT^ values for the same series of antioxidants were determined by employing the free Molinspiration software [76], which permits estimations of *P*_W_^OCT^ values based on group contributions, and these are also indicated. Details on the use of the Molinspiration software can be found elsewhere [76].

*P*_W_^O^ values for C1–C8 derivatives of gallic acid (Table 5) are ≈ 60–270 times lower than those reported in n-octanol/water mixtures (*P*_W_^OCT^). This important difference between the *P*_W_^OCT^ and *P*_W_^O^ values indicates that C1–C8 gallate esters are much more soluble in 1-octanol than in olive oil, and it indicates that *P*_W_^OCT^ values cannot be used to predict the hydrophobicity of gallates in edible oils. From the molecular point of view, the main polar group in olive oil is the glycerol ester with three ester groups, whereas octanol has a hydroxyl group. Thus, the hydrogen bond donating capacity of octanol is probably responsible for the greater solubility in octanol of AOs.

**Figure 3:**
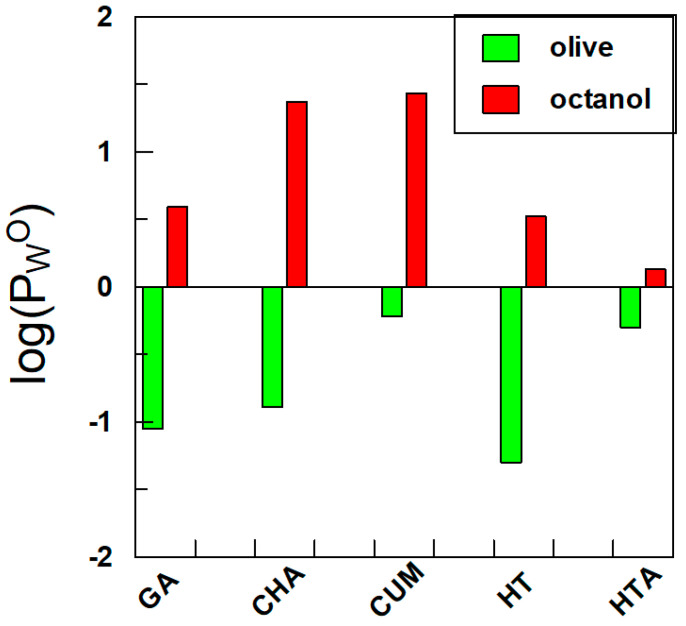
Comparison between the experimentally determined *P*_W_^O^ values in oil–water systems and the theoretical *P*_W_^Octanol^ values for a series of antioxidants with different molecular structures. GA: gallic acid, CHA: catechin, CUM: *p*-coumaric acid, HT: hydroxytyrosol, HTA: hydroxytyrosol acetate.

Figure 3 compares the partition constants in olive-oil mixtures with those in octanol–water for a number of antioxidants with different reactive moieties. The disparity of values between the experimentally determined *P*_W_^O^ values in oil–water systems compared to the theoretical *P*_W_^Octanol^ confirms that *P*_W_^O^ values cannot be predicted from *P*_W_^Octanol^ and need to be determined for each antioxidant and oil.

### 4.3. Determining the Partition Constants of Antioxidants in Intact O/W Emulsions

Binary oil–water mixtures are thermodynamically unstable, but they can be stabilized kinetically, sometimes for long time periods, by adding surfactants [79]. Thus, when oil and water are shaken gently in the presence of surfactants, micrometer-sized (0.1–100 μm) spherical droplets of one liquid dispersed in the other are obtained. Surfactants adsorb in the surface of the freshly formed emulsion droplets during the homogeneization process, creating a three-dimensional interfacial region of 2–20 nm thick between the water and oil regions that stabilizes kinetically the emulsion. Unless the droplet size is smaller than ≈1 μm or large concentrations of surfactant are employed (Φ_I_ > 0.07), the volume of the interfacial region does not contribute significantly to the total volume of an emulsion, being less than 5–7% of the total emulsion volume. In spite of the small volume of the interfacial region, determining the distribution of antioxidants (or that of other emulsion components) in emulsions is not a trivial task, because experiments need to be performed in the intact emulsions to avoid disruption of the existing equilibria and because interfacial concentrations can be substantially different from the stoichiometric concentrations [40,64,65,80,81]. The first proposed methods for determining the distributions of antioxidants were based on the breakdown of the emulsion followed by the separation of each of the phases by centrifugation and/or filtration and determination of the antioxidant concentrations in each phase by, for example, high-performance liquid chromatography (HPLC) [82,83].

Stöckmann and Schwarz [84] combined ultrafiltration and dialysis techniques together with a mathematical model to estimate the distribution of some phenolic derivatives in emulsions. Unfortunately, the ultracentrifugation technique allows for distinction between the aqueous and interfacial regions but not between the interfacial and oil regions. Thus, results were somewhat biased and reflect that any determination of the partitioning of the antioxidants needs to be performed in the intact emulsions.

**Scheme 10:**
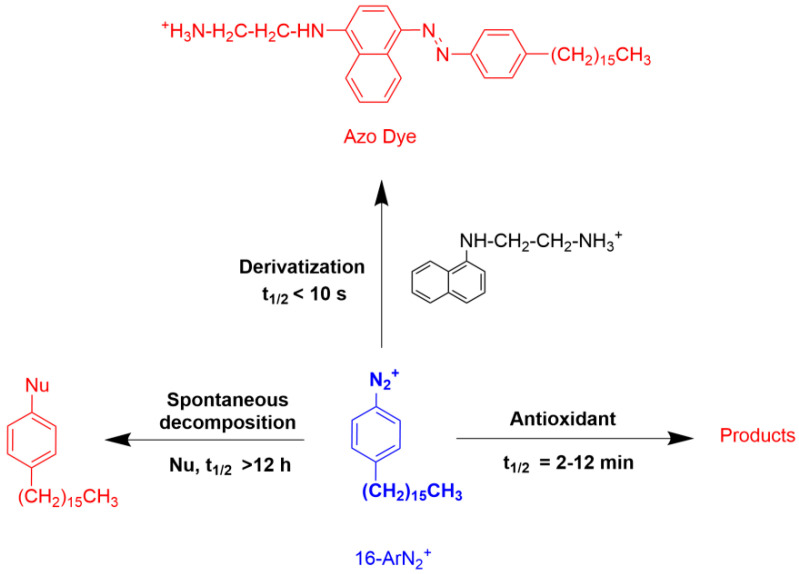
Main reactions involved in the use of the chemical probe 16-ArN_2_^+^ in emulsions to determine antioxidant distributions. The typical half-lives, *t*_1/2_, of the reactions are indicated.

A conceptually different approach was developed based on the use of a specially synthetized chemical probe, 4-hexadecylbenzenediazonium, 16-ArN_2_^+^, ion, as shown in Scheme 10. For determining the antioxidant concentrations in each phase, focus was placed on developing methods to determine the partition constants of the antioxidants between the oil–interfacial and water–interfacial regions in the intact emulsions [44,45,85,86]. The approach is grounded in the application of pseudophase kinetic models, which were first developed to model chemical reactions in association colloids [44,85]. To model chemical reactions in emulsions, the same logic used to interpret chemical reactions in association colloids can be employed. Basically, two assumptions are required: (1) antioxidants are distributed thermodynamically, so that their distribution is controlled by their relative solubility in each region, and (2) their distribution is fast in the time scale of thermally activated chemical reactions, so that emulsion components, and particularly the reactants, are at dynamic equilibrium.

The reasons for choosing this probe were various. (1) There is a solid background on the application of ArN_2_^+^ ions as chemical trapping molecules to probe the interfacial composition of colloids. Arenediazonium ions react with virtually all antioxidants, making them excellent candidate to determine their distributions. (2) The rates for their spontaneous decomposition are very slow in aqueous acid, in the dark, and in the absence of reductants, and hence do not interfere with the reaction with antioxidants. (3) The variation in their concentration with time in opaque systems can be monitored electrochemically or, for instance, by employing a derivatization method. (4) The specific design of 16-ArN_2_^+^, with a long hexadecyl hydrophobic tail that makes it water insoluble, and the cationic character of the reactive group, which makes it oil insoluble, locates the reactive, -N_2_^+^, in the interfacial region of emulsions, where it reacts with the antioxidants available. Details of the method can be found elsewhere [44,45,85].

By taking into consideration the corresponding kinetic equations, the AO and 16-ArN_2_^+^ mass balances, and the definitions of the partition constants, the relationships 12–15 have been derived. Details of the mathematical procedures are given elsewhere [44,85].

(12) $$k_{\mathrm{obs}}=k_{2}[AO_{T}]=\frac{[AO_{T}]k_{I}P_{O}^{I}P_{W}^{I}}{\Phi_{O}P_{W}^{I}+\Phi_{I}P_{O}^{I}P_{W}^{I}+\Phi_{W}P_{O}^{I}}$$

(13) $$k_{\mathrm{obs}}=\frac{k_{I}{\left[\mathrm{AO}\right]}_{T}P_{W}^{I}}{\Phi_{I}P_{W}^{I}+\Phi_{W}}$$

(14) $$k_{\mathrm{obs}}=\frac{k_{I}{[\mathrm{AO}]}_{T}P_{O}^{I}}{\Phi_{I}P_{O}^{I}+\Phi_{O}}$$

(15) $$k_{\mathrm{obs}}=\frac{k_{I}{\left[\mathrm{AO}\right]}_{T}}{\Phi_{I}}$$

Equation (12) is the general equation that shows the dependence of *k*_obs_ on the surfactant volume fraction, water and oil volume fractions, and the medium effect of the emulsion interface.

Emulsions are opaque, and special methods were developed to measure rate constants in the intact emulsions. Briefly, *k*_obs_ values can be obtained by employing cyclic voltammetry or by employing a derivatization method that traps unreacted 16-ArN_2_^+^ via the C-coupling reaction with a suitable coupling agent *N*-(1-naphthyl) ethylenediamine(NED), as shown in Scheme 10. The reaction of ArN_2_^+^ with NED is much faster than both the spontaneous decomposition and the reaction with antioxidants, which is a necessary requirement to get reliable values for the observed rate constants, *k*_obs_, between ArN_2_^+^ and the antioxidant. Details on the methods can be found elsewhere [44,45]. In practice, the partition constants are determined by keeping the ratio Φ_O_/Φ_W_ constant, so that plots of the variations of *k*_obs_ versus Φ_I_ at constant Φ_O_/Φ_W_ are obtained. In addition, a value for the partition constant of the AO between oil and water in the absence of surfactant, *P*_W_^O^, is needed. An alternative method requires obtaining plots of *k*_obs_ versus Φ_I_ at different Φ_O_/Φ_W_ ratios, but this approach is not usually employed because it is time consuming and enormous experimental efforts are required.

**Figure 4:**
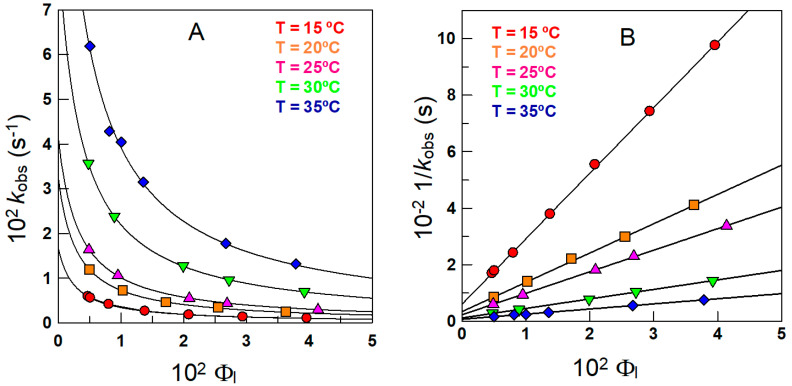
Illustrative variations of *k*_obs_ (**A**) and 1/*k*_obs_ (**B**) vs. Φ_I_ for caffeic acid (CA) at different temperatures. The solid lines are the theoretical curves obtained by fitting the experimental data to Equation (13) and to its reciprocals. Reprinted from ref. [66] with permission, copyright Royal Society of Chemistry.

Equations (13)–(15) are simplifications of this general kinetic equation that can be made for limiting situations where (i) the reactant is oil insoluble (i.e., very hydrophilic), Equation (13), (ii) water insoluble (i.e., very hydrophobic), Equation (14), or (iii) both oil and water insoluble, Equation (15). Equations (12)–(15) predict that at constant temperature, acidity, [AO_T_], and at a constant Φ_W_:Φ_O_ ratio, *k*_obs_ must decrease asymptotically with increasing Φ_I_, as illustrated in Figure 4, where plots of the typical variations of *k*_obs_ and 1/*k*_obs_ vs. Φ_I_ for caffeic acid (CA) under different experimental conditions are shown.

Bearing in mind the corresponding mass balances, the concentrations of the antioxidant in a particular region can be obtained. Equations (16)–(18) illustrate the effective concentrations in the interfacial region of the emulsion relative to the stoichiometric concentrations for polar (hydrophilic) antioxidants, antioxidants of moderate hydrophobicity, and very hydrophobic (apolar) antioxidants, respectively.

(16) $$\frac{{(\mathrm{AO}}_{I})}{{[\mathrm{AO}}_{T}]}=\frac{P_{W}^{I}}{\Phi_{W}{+P}_{W}^{I}\Phi_{I}}$$

(17) $$\frac{\left({\mathrm{AO}}_{I}\right)}{\left[{\mathrm{AO}}_{T}\right]}=\frac{P_{O}^{I}P_{W}^{I}}{\Phi_{O}P_{W}^{I}+\Phi_{I}P_{O}^{I}P_{W}^{I}+\Phi_{W}P_{Ó}^{I}}$$

(18) $$\frac{\left({\mathrm{AO}}_{I}\right)}{\left[{\mathrm{AO}}_{T}\right]}=\frac{P_{O}^{I}}{\Phi_{O}+\Phi_{I}P_{O}^{I}}$$

Equations (16)–(18) predict that (1) the interfacial concentration depends on both the partition constants *P*_W_^I^ (which, in turn, may depend on pH) and *P*_O_^I^, the oil-to-water o/w ratio, and the surfactant volume fraction Φ_I_. (2) At constant pH and o/w, an increase in Φ_I_ decreases the (AO_I_)/[AO_T_] ratio approaching a plateau as all the AOs are dissolved in the interfacial region, and (3) at a fixed Φ_I_, an increase in the o/w ratio works in opposite directions for hydrophilic and hydrophobic AOs. An increase in the relative amount of oil leads to an increase in the interfacial concentration of the hydrophilic AOs (e.g., gallic acid) while it decreases that of the hydrophobic (lauryl gallate). The effects of a change in the o/w ratio on the partitioning of AOs of intermediate polarity are not so obvious because the interfacial concentration also depends on the particular values of *P*_W_^I^ and *P*_O_^I^.

**Figure 5:**
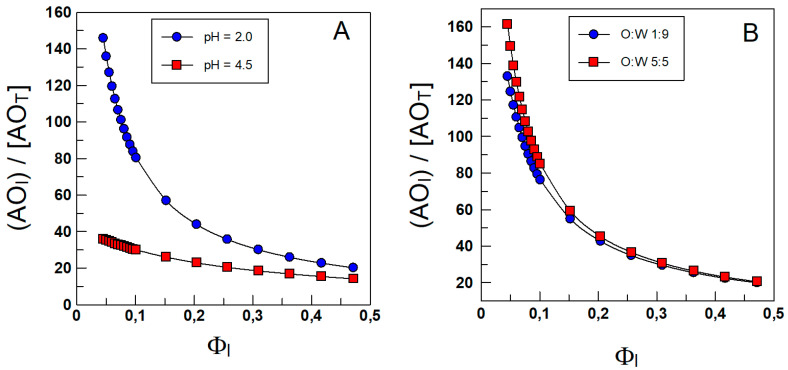
(**A**) Illustrative theoretical variations of the ratio (AO_I_)/[AO_T_] with Φ_I_ for gallic acid (pKa = 4.5) and partition constants of *P*_W_^I^ = 300 at pH = 2 and *P*_W_^I^ (app) = 30 at pH = 4.5. (**B**) Effects of the oil-in-water (o/w) ratio on the variation of the ratio (AO_I_)/[AO_T_] with Φ_I_ for gallic acid at pH = 2 (*P*_W_^I^ = 300). In both cases, theoretical (AO_I_)/[AO_T_] values were obtained by employing Equation (16).

Figure 5A illustrates the variations of the ratio (AO_I_)/([AO_T_] with Φ_I_ for gallic acid, which is a very hydrophilic antioxidant with a partition constant of *P*_W_^I^ = 300 at pH = 2 (neutral form), and for the same antioxidant when it was ionized (*P*_W_^I^ (app) = 30 at pH = 4.5), according to Equation (16). At the lowest surfactant volume fraction employed, Φ_I_ = 0.005, the interfacial concentration of the neutral AO is ≈150 times higher than that of the stoichiometric concentration. However, upon ionization, the interfacial concentration decreases ≈4 fold at the same emulsifier volume fraction. At any pH, an increase in the surfactant concentration leads to an effective dilution of the AO in the interfacial region. For instance, for the neutral AO, (AO_I_)/[AO_T_] decreases ≈6 fold on going from Φ_I_ = 0.005 to Φ_I_ = 0.5; meanwhile, when ionized, the decrease in (AO_I_)/[AO_T_] is only ≈2 fold, because a portion of the antioxidant is located in the aqueous region as a consequence of ionization.

A change in the o/w ratio leads to modest changes in the interfacial concentrations of gallic acid, as shown in Figure 5B, which illustrates the effects of a change in the oil to water ratio at constant pH = 2. Interfacial AO concentrations are as much as ≈165 times higher than that of the stoichiometric concentration in 5:5 emulsions, and they decrease 20% upon decreasing the content of oil in the emulsion (≈135 times higher than that of the stoichiometric concentration).

Results in Figure 5 show that an increase in the surfactant volume fraction leads to an effective dilution of AOs in the interfacial region, decreasing 2–6 fold on going from Φ_I_ = 0.005 to 0.05 (10 fold increase) depending on the acidity. There is also the remarkable and significant effect that the acidity of the medium has on the interfacial concentrations of the antioxidants when phenolic acids are involved.

Results in Figure 5 also highlight the importance of determining the effective interfacial concentrations to correctly predict antioxidant efficiency because as we will show in the next section, the partitioning of antioxidants in the interfacial region correlates directly with the antioxidant efficiency. Indeed, the relationships between the hydrophilic–lipophilic balance (HLB) of antioxidants (AOs) and their distribution and efficiency in emulsions are not fully understood, and more work is still needed because prediction of the distribution of AOs on the basis of the molecular characteristics and of the environmental conditions is still far from being completely understood.

### 4.4. Role of Motion of Reactants in Fluid Emulsions: Dynamic Equilibrium

In a recent review, Laguerre et al. [87] raised the question of whether lipid oxidation reactions in emulsions are kinetically or diffusion controlled—that is, if the transport of reactants between the various domains of emulsions is rate-limiting. In connection with this point, they also put forward the idea that “partitioning studies are only pictures of a process, not a dynamic description of its evolution. It is like trying to understand a movie by looking only a few frames”. In our opinion, this is a somewhat distorted vision of the phenomena, as partitioning experiments do reflect the dynamic of motion of the molecules, going back and forth until an equilibrium position is reached [88], and thus are far from being static pictures [69,71,75].

For decades, ground-state thermal reactions were employed in combination with pseudophase models to provide insights into the properties of the various domains of colloidal systems [27,44,45,89,90,91,92]. The assumption under the mathematical treatments to interpret chemical reactivity in colloidal systems lies in treatments of reactivity in homogeneous systems in which usually diffusivity is orders of magnitude faster than the chemical reaction of interest, so that the distribution of reactants are in dynamic equilibrium and controlled by Brownian motion. Bravo-Díaz et al. [93] applied the ideas underlying the Acree–Curtin–Hammet principle [94] and the theory of diffusion-controlled reactions [95,96] to reactions taking place in phases of different viscosities, concluding that if the rate constants for molecular diffusion are >10^3^ greater than the second-order rate constant for the reaction between reactants, the molecular transport is much too fast to be rate determining [44,45,93]. These are the normal reaction conditions in fluid solutions [96] and even in food systems [97,98].

An alternative approach is to express reactivity in terms of molecular diffusion. After bulk mixing is complete, the rate of any bimolecular reaction depends on their diffusion rates. If they are near the diffusion-controll limit, the reaction is under *microscopic diffusion control*, and the rate of the reaction is limited by the encounter frequency of reactants. If the observed rate constant is equal to that of the chemical reaction, then the reaction is under *microscopic activation control*, i.e., the rate of the reaction depends on its activation energy [93,96].

According to the Smoluchowsky´s theory, the size of the molecules has little effect on diffusion coefficients because large molecules move more slowly than the smaller ones but present larger target areas for encounters with other molecules, and the two effects approximately cancel each other [93,96]. Viscosities of food fluids can be 1–10^4^ orders of magnitude greater than the viscosity of water, and the corresponding diffusion rate constants should be 10^4^ times lower than in water [97]. Even in extremely thick emulsions such as mayonnaise, whose viscosity is 10^4^–10^5^ times higher than that of water, the diffusion of reactants is 10^4^–10^5^ times faster than the rates of common thermal reactions. In other words, the chemical reaction in the emulsion would have to be one in the microsecond time scale to be affected by the bulk viscosity of mayonnaise. Further details can be found elsewhere [93,96,97].

Even though the lipid oxidation reaction is a very complex reaction comprising numerous by-side reactions, it is well demonstrated that once the first initiating radical is formed, the rate-limiting reaction is that between the formed peroxyl radical LOO^•^ and the unsaturated lipid [30,50]. The activation energy of this reaction is 40–100 kJ mol^−1^ [93,97], which is much higher than those typically found for diffusion-controlled reactions [96,99], and therefore, the reaction is under *microscopic activation control*. Of course, this does not invalidate the fact that any of the by-side reactions that may take place during the propagation step may be under *microscopic diffusion control*, so that the rate of this particular step is limited by the encounter frequency of the reactants [29,93].

To sum up this section, the chemical kinetic method shown, and the partitioning studies carried out are not static pictures but fully describe the dynamic distribution of the antioxidants between the oil, interfacial, and aqueous regions of model food emulsions. As we will see in the next section, the method developed based on the use of thermally activated chemical reactions in systems under dynamic equilibrium is the only approach to date that permits quantifying the effective concentrations of antioxidants in the various regions of the emulsions. These concentrations, and particularly, those of the interfacial region, are crucial to interpret and predict the efficiency of antioxidants, and the rates of the inhibition reaction depend not only on the value of the rate constant, which reflects the medium properties, but also on the effective concentration of the reactants at the reaction site, which is different from the stoichiometric concentration in the emulsifier system, and it allows explaining from a molecular point of view the variations in the observed efficiencies of antioxidants, including the so-called cut-off effect [40,44,45,65,80,100].

### 4.5. Relationships between Partitioning and Efficiency: Concentration Effects

Antioxidants may react with peroxyl radicals through various mechanisms; the most important are the combination of hydrogen atom transfer (HAT) and single electron transfer (SET) mechanisms. The rate of this reaction depends on the conformational, electronic, and geometrical features of polyphenols, as well as the solvent properties of the reaction site.

In multiphasic systems, the proper choice of efficient AOs is puzzling, because their efficiency is governed by both their rates of scavenging free radicals and their effective concentrations at the reaction site, which are controlled by their partitioning in the emulsified system. The foundation for grasping the substituent and structural effects on the antioxidant activities of phenols rests on the quantitative kinetic studies of absolute rate constants for hydrogen atom transfer from substituted phenols to peroxyl radicals. Detailed reviews in bulk systems were published, and the interested reader is referred to them [42,50,101], but key aspects relating the structural features of AOs to their efficiencies in emulsified systems are not well known, in part, because there is not a direct relationship between the chemical structure of the AOs and their efficiency. For example, an increase in the length of the alkyl chain of AOs changes both their relative solubility in the oil, water, and interfacial regions of the emulsion and their hydrophilic–lipophilic balance (HLB) [44,66,102], and it may also modify the AO oxidation/reduction potentials [103]. Thus, in addition to the effects of structural modifications on the aromatic rings of polyphenols, some control on a number of other parameters—at least the effective concentrations of antioxidants at the reaction site—need to be taken into consideration to predict and improve their efficiency.

The reactivity of antioxidants depends on (a) the number and position of -OH groups attached to the aromatic ring; and (b) the number, position, and nature of the functional groups grafted to the phenolic ring and their possibility of conjugation, electronic delocalization, and resonance effects with the aromatic ring. The hydrogen atom-donating ability of phenols strongly depends on the nature and position of substituents in the aromatic ring of polyphenols. Electron-donating substituents at the *ortho* and/or *para* positions of the –OH group decrease the phenolic O−H bond dissociation enthalpy, increasing the reaction rates with peroxyl radicals and thus making antioxidants more efficient.

Consequently, determining the most efficient AO for a particular emulsion system remained, until recently, an unsolved problem that has been overcome with the application of pseudophase kinetic methods to determine the distributions of antioxidants in the intact emulsions.

The oxidative state of a sample can be monitored by employing a variety of methods that mostly can be grouped on the basis of the control parameter employed: consumption of oxygen, loss of initial substrates, formation of primary (hydroperoxides) and secondary (aldehydes, ketones, hydrocarbons, etc.) oxidation products, and the formation of free radicals. These methods are usually employed under accelerated conditions such as, for example, by increasing the temperature (40–140 °C), by adding a metallic catalyst (1–100 ppm), by increasing the oxygen pressure (3–165 psi), or by adding a radical initiator, and then, an appropriate end point is chosen to determine the appropriate levels of oxidative deterioration [30,49,58,104,105,106,107]. The choice of the most appropriate method and experimental conditions is critical in interpreting the obtained results.

**Figure 6:**
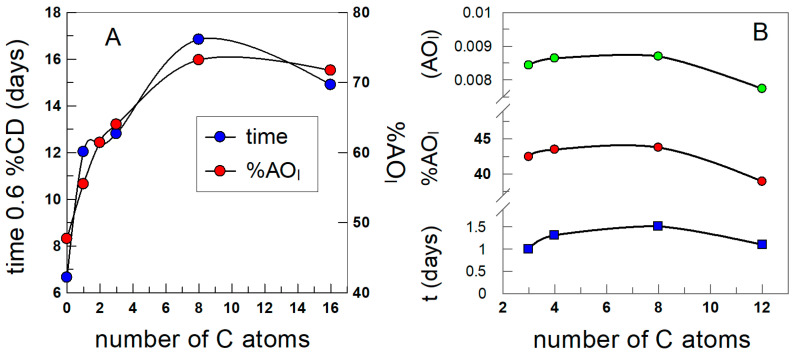
Direct correlation between the antioxidant efficiency (time to reach a selected percentage of conjugated dienes) and the percentage of antioxidant in the interfacial region for (**A**) caffeic acid and its derivatives and (**B**) gallic acid and its derivatives. (**A**) Reproduced from reference J Sci Food Agric (2016); (**B**) Reproduced from reference Food & Function, 2018, 9, 4429–4442.

Inspection of the effects of the alkyl chain lengths for several series of homologous AOs on their efficiency demonstrate that is parabolic-like, with a maximum at an intermediate (C_4_–C_12_) chain length (e.g., in Figure 6, a maximum at an octyl caffeate was found) [44,68,108,109,110]. This parabolic dependence of AO efficiency upon chain length, known as the “cut-off” effect [109,111,112], was reported for the first time more than a century ago when describing both the chemical and biological activity of series of homologous AOs, and it has been now commonly observed for a series of various antioxidants [111,113,114,115,116,117]. Several hypotheses have been put forward to rationalize the cut-off effect on the antioxidative and biological activities of a series of antioxidants, including the following: (a) changes in reactant diffusivity; (b) changes in the self-aggregation ability of the long-chain derivatives; (c) differential solubility of the antioxidants in the interfacial region; and (d) differences in reactivity could be accounted for changes in physical properties of the reactants, e.g., chain length induced changes in reactivity orientation in the interfacial region. Unfortunately, none of them was proven experimentally.

The approach developed by Romsted, Bravo, and coworkers allowed the determination of the antioxidant distribution for different homologous series of AOs (see Section 4.1) and its correlation with the antioxidant efficiency [44]. In a series of papers, concerning AO efficiency in nonionic emulsions for different AOs (gallic acid, caffeic acid, hydroxytyrosol, chlorogenic acid) and their esters of various chain lengths, they provided physical evidence that the efficiency of antioxidants depends on their interfacial concentrations, which can be modulated by increasing the hydrophobicity of the AOs and by employing the minimum amount of surfactant necessary to stabilize the emulsion. The authors also showed that changes in the oil-to-water ratio can also be used to optimize interfacial concentrations of very hydrophobic or very hydrophilic antioxidants, but not those of antioxidants of intermediate hydrophobicity [77].

Figure 6 is representative and shows the parallel variations between the efficiency, the percentage of antioxidant in the interfacial region, and the effective interfacial concentrations of a series of homologous antioxidants bearing the same reactive moiety but of different hydrophobicity.

A number of physicochemical properties (hydrophobicity, degree of ionization, etc.) of antioxidants and the environmental conditions such as the nature of the oil, the oil/water ratio, the hydrophilic–lipophilic balance (HLB), emulsifier electrical nature and concentration, and temperature may affect significantly the distribution of AOs between the oil, interfacial, and aqueous regions of the emulsions, and hence modify their interfacial concentrations [40,64,65,81]. For instance, some researchers [118] pointed out that the acylation of antioxidants may not necessarily improve their efficiency in bulk oils and emulsions, assuming that the lengths of the lipohilized antioxidants they employed were above the “cut-off” maximum, i.e., the length of the alkyl chain was too long to allow antioxidants to be located in the interfacial region. In a series of studies, the same authors concluded that the lipid oxidation reaction was influenced by the emulsion system [119], pointing out that the presence of impurities such as free fatty acids in their systems, or some kind of interaction with the emulsifier (citrem) employed, may be the cause of the low efficiency of some of the investigated antioxidants. They also raised the question of whether “interactions” with other components may prevent them from acting as antioxidants. Qiu et al. [120] made some partitioning experiments in fish oil-enriched milk systems and proposed that “interactions” between antioxidants esterified with short and medium alkyl chain lengths may lead to “synergistic” effects, which eventually could change their partitioning or localization at the interface. Unfortunately, no detailed description of such interactions or quantization of the synergistic effects was provided to the best of our knowledge, which was probably because of the enormous complexity of milk as a food system.

Laguerre et al. [87] pointed out the putative hypothesis that an excess of emulsifiers may affect the partitioning of antioxidants or pro-oxidants. This certainly adds some complexity to interpreting experimental results in emulsions. However, recent results by Costa et al. [64,65] suggest that the onset of lipid oxidation and its inhibition by antioxidants is not altered in emulsions with a deficit or excess of surfactant, and therefore, further work to prove or discard the hypothesis is required. Sorensen et al. [121] pointed out the possibility that endogenous antioxidants (tocopherol) present in emulsions prepared with fish oil/rapeseed oils may affect their antioxidant activity, attributing their observations to emulsifier–antioxidant and antioxidant–antioxidant “interactions” in the emulsions. No description of the nature of such interactions was made, but this brings up the interesting point of whether there are such potential interactions with endogenous antioxidants (for example, if added antioxidants are synergistic or antagonist with endogenous tocopherol), and it also highlights the complexity of interpreting results in emulsified systems compared to bulk ones. The same authors highlight the importance of evaluating the antioxidant efficiency in each emulsion before selecting the best antioxidants for an optimal protection against lipid oxidation [121]. We fully agree with them, and for this reason, we believe that it is necessary to attempt to quantitatively interpret all observations, not just limited to mere descriptions of the bulk experimental observations, making more relevant those studies aimed to systematically analyze the effects of the various parameters controlling the inhibition of the lipid oxidation reaction under different experimental conditions.

Finally, we should not forget the location of the peroxyl radicals, which are crucial partners in the inhibition reaction. Aliaga et al. [122,123,124,125,126] approached the problem by employing a series of nitroxide radical derivatives and probes of variable hydrophobicity to investigate the importance of the distribution and orientation of the probe and substrate in microheterogeneous media, concluding that the orientation and buoyancy ability of radicals may also play a crucial role in the fate of the inhibition reaction. Therefore, quantification on how antioxidants and lipid radicals distribute in emulsions and their relative orientation at the interface is central for understanding and ranking their efficiencies in inhibiting lipid oxidation.

## 5. Conclusions and Future Perspectives

Lipid emulsions are essential components of a non-protein energy source in parenteral nutrition. However, soybean oil lipid emulsions, due to their richness in omega-6 PUFA such as linoleic acid, increase lipid peroxidation and inflammation. It has been also suggested that these emulsions can cause an increase in intra-ventricular hemorrhage, retinopathy of prematurity, and bronchopulmonary dysplasia development by generating oxidative damage [127]. The search for alternatives has gone mainly by replacing the type of oil in these formulations. The newest lipid solutions contain mixtures of SO, medium-chain triglycerides, olive oil, and fish oil, as sources of essential fatty acids, energy, monounsaturated fatty acid (MUFA) and omega-3 fatty acids. These new-generation formulations have been added with tocopherol in order to increase their oxidative stability, and it has been suggested that these new lipid solutions decrease the parenteral nutrition related problems by causing less liver damage, less oxidative damage, and less lipid peroxidation.

Current research evidence, mainly coming from the epidemiologic cohort and case studies, also suggests that the long-term consumption of polyphenol-rich foods helps to protect against a variety of diseases, including cancer as well as cardiovascular and neurodegenerative processes. Changes in the composition and/or preparation of the lipid-based system result in the preparation of a range of emulsions with new properties, all of them having (in principle) a great potential in the delivery of polyphenols or other bioactive compounds. Consequently, many emulsion-based delivery systems for polyphenols have been well-established. Therefore, the use of unsaturated fatty acids as the oil phase in delivery systems would provide a valuable energy source for patients and at the same time an excellent vehicle to deliver amphiphilic and highly hydrophobic drugs.

However, the location and performance of the antioxidants or other bioactives in the delivery system is crucial for their practical applications. In contrast to solid particles, emulsion droplets have deformable surfaces and emulsion components, and other added bioactive compounds diffuse and adsorb and desorb from the continuous and disperse liquid regions to the interface region. Thus, the efficiency of antioxidants in inhibiting the lipid oxidation reaction can be compromised because the added polyphenols distribute between different regions according to their polarity, and therefore, research on methods to determine the location of antioxidants and their distribution is crucial and of great importance. It is hoped that the information presented in this review will enable understanding which factors are driving the distributions of antioxidants to understand how lipid oxidation can be controlled and effectively blocked. Lipid oxidation and its inhibition by antioxidants constitute a set of chemical reactions that need to be analyzed to correctly interpret their behavior. This need of proper kinetic understanding and the determination of crucial kinetic parameters drove us to develop a new approach based on the structural similarities of microemulsions and emulsions that let us determine the distribution of AOs in these systems. It probably constitutes a perfect example on how typical physical organic chemistry approaches can be employed to get important information on processes central to food chemistry. It should inspire researchers to find different approaches to investigate in detail the lipid oxidation reactions to improve our basic knowledge and, in practice, to control and minimize their harmful effects. For example, recent research was focused on investigating the location, orientation, and partitioning of radicals rather than the antioxidants, developing new approaches to obtaining a deeper understanding of mechanism(s) of lipid peroxidation in opaque emulsions [122,123,124,125,126].

Nevertheless, much work is still necessary in areas such as pharmacy, medicine, biology, etc., where emulsions are widely employed in, for example, in optimizing drug distribution, drug delivery, and oxidative stability of parenteral formulations. Present food challenges include the development of edible delivery systems to encapsulate, protect, and release bioactives and functional lipophilic constituents (e.g., ω-3 lipids) and to understand a number of factors (distribution, pH, interfacial charge, etc.) that are behind the antioxidant, pro-oxidant, and synergistic effects observed during the use of potential antioxidant molecules on the chemical stability of emulsions. Control of the effects of these parameters on the partitioning of antioxidants will permit developing guidelines not only for selecting the most efficient AO for a particular application but also for the successful design of alternate, effective antioxidant solutions.

## Figures and Tables

**Table 1:** Chemical structures and common names of some fatty acids.

Chemical Structure	Number C Atoms	Omega Name	Common Name	Systematic Name
**saturated fatty acids**				
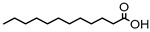	12	12:0	Lauric	Dodecanoic
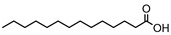	14	14:0	Myristic	Tetradecanoic
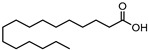	16	16:0	Palmitic	Hexadecanoic
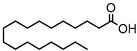	18	18:0	Stearic	Octadecanoic
**unsaturated (*cis*) fatty acids**				
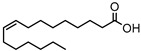	16	16:1	Palmitoleic	9-Hexadecenoic
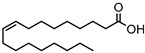	18	18:1 (w-9)	Oleic	9-Octadecenoic
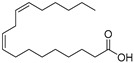	18	18:2 (w-7)	Linoleic (LA)	9, 12-Octadecadienoic
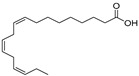	18	18:3 (w-3)	α-Linolenic (ALA)	(all-cis-9,12,15) Octadecatrienoic
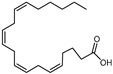	20	20:4 (w-6)	Arachidonic	5,8,11,14-Eicosatetraenoic
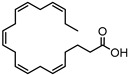	20	20:5 (w-3)	Eicosapentaenoic (EPA)	5,8,11,14,17-Eicosapentaenoic
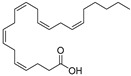	22	22:5 (w-6)	Docosapentanoic (DPA)	4,7,10,13,16- Docosapentanoic
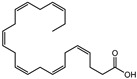	22	22:6 (w-3)	Docosahexaenoic (DHA)	cis-docosa-4,7,10,13,16,19-hexaenoic acid

**Table 2:** Fatty acid composition (%) of various oils commonly employed in parenteral lipid emulsions [10].

	Soybean	Safflower	Olive	Fish
Oleic acid (n-9)	25	15	85	0
Linoleic acid (n-6)	50	77	4	1–3
Linolenic acid (n-3)	10	0	< 1.5	1–5
EPA	0	0	0	5–14
DHA	0	0	0	5–27

**Table 3:** Main reactive oxygen species (ROS) [35].

Non-radical	Hydrogen peroxide	H_2_O_2_
	Singlet oxygen	^1^O_2_
Radicals	Superoxide anion	O_2_^− •^
	Hydroxyl	HO^•^
	Lipid peroxyl	ROO^•^
	Lipid alkoxyl	RO^•^

**Table 4:** Types of antioxidant defense in biological systems [4,60].

Primary	Vitamin E (α-tocopherol)
	Vitamin C (ascorbic acid)
	Carotenoids
	Polyphenols
Secondary	Superoxide dismutase
	Glutatione peroxidase
	Transition metals chelators
	Catalase (Fe)

**Table 5:** Values of the partition constant *P*_W_^O^ (olive oil–water) and *P*_W_^OCT^ (octanol–water) for several gallic acid derivatives in binary mixtures (determined by employing the Molinspiration software [76], see text for details). (1) Data from ref. [77] (2) Value from Huang et al. [78].

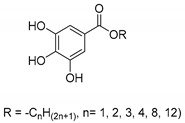		C1	C2	C3	C4	C8	C12
	***P*** **_W_** **^O^**	0.05 ^1^	0.20^1^	0.86 ^1^ 0.85 ^2^	3.04^1^	75 ^1^	---
	***P*** **_W_** **^OCT^**	7.10	17	50	195	2.0 × 10^4^	2.13 × 10^6^

## Data Availability

Data supporting reported results can be obtained from any masthead.
